# 人源化BCMA CAR-T细胞挽救治疗鼠源BCMA CAR-T细胞治疗后再进展的难治多发性骨髓瘤二例

**DOI:** 10.3760/cma.j.issn.0253-2727.2021.06.010

**Published:** 2021-06

**Authors:** 蕊 崔, 平 李, 青 李, 娟 穆, 怡丽 蒋, 嫣雨 江, 琦 邓

**Affiliations:** 1 天津市第一中心医院血液科，南开大学医学院 300192 Department of Hematology, Tianjin First Central Hospital, School of Medicine, Nankai University, Tianjin 300192, China; 2 德州市人民医院血液科 253000 Department of Hematology, Dezhou People's Hospital, Shandong 253000, China

**Keywords:** 多发性骨髓瘤, 人源化, 嵌合抗原受体T细胞, 安全性, 疗效, Multiple myeloma, Humanized, CAR-T, Safety, Efficacy

## Abstract

**目的:**

探讨人源化B细胞成熟抗原（BCMA）嵌合抗原受体T细胞（CAR-T细胞）治疗鼠源BCMA CAR-T后疾病再进展的难治性多发性骨髓瘤（RRMM）患者临床疗效及安全性。

**方法:**

采集两例患者自体外周血单个核细胞，制备BCMA CAR-T细胞，FC方案（氟达拉滨+环磷酰胺）预处理后分别予鼠源/人源化BCMA CAR-T细胞输注。输注后监测CAR-T细胞扩增、细胞因子变化及不良反应。体外试验检测鼠源/人源化BCMA CAR-T转染效率、对MM细胞株的杀伤活力及炎症细胞因子释放水平。

**结果:**

例1及例2输注鼠源CAR-T后3个月分别为完全缓解（CR）及疾病稳定（SD）。16个月及18个月后出现疾病再进展，且例1出现髓外病变，输注人源化BCMA CAR-T细胞挽救治疗后，分别达到部分缓解（PR）及非常好的部分缓解（VGPR）的疗效，例1髓外病变4个月消失。两例患者在人源化BCMA CAR-T细胞治疗期间，CAR-T细胞体内扩增峰值、体内持续时间均较鼠源输注期间水平升高。人源化BCMA CAR-T治疗期间IL-6、IL-8、IFN-γ、IL-10及TNF-α峰值高于鼠源CAR-T峰值。两例患者输注鼠源CAR-T期间细胞因子释放综合征（CRS）均为1级，无神经系统毒性（ICANS）；人源化CAR-T治疗例1 CRS为3级，ICANS为2级，支持对症治疗后好转，例2 CRS 2级，无ICANS发生。体外试验证实48 h效靶比为1∶1时，人源化BCMA CAR-T、鼠源CAR-T细胞分别与例1、例2患者共培养，BCMA^+^肿瘤细胞残余比例分别为（17.38±5.18）％对（28.27±4.58）％、（13.25±1.62）％对（22.77±1.77）％，人源化BCMA-CAR-T对原代MM的细胞毒作用优于鼠源CAR-T细胞（*P*<0.001），且IFN-γ、TNF-α及IL-6释放水平均高于鼠源CAR-T细胞（*P*值均<0.001）。

**结论:**

鼠源BCMA CAR-T治疗后复发进展的RRMM患者再次输注人源BCMA CAR-T可能有效且安全性可控。

## Abstract (en)

**Objective:**

To observe the efficacy and safety of humanized anti-BCMA chimeric antigen receptor modified（BCMA CAR）-T cell therapy after disease progression with their murine BCMA CAR-T cell therapy in patients with relapsed/refractory multiple myeloma （MM）.

**Methods:**

Study participants underwent leukapheresis to collect T cells for BCMA CAR-T manufacturing. Patients were pretreated with intensive chemotherapy（fludarabine combined with cytarabine）before CAR-T therapy. Adverse events（AEs）, CAR DNA expansion, and cytokine were monitored. In vitro, transfection efficacy, specific cytotoxicity, and inflammatory response were detected when co-cultured with effector and target cells.

**Results:**

Patient（PT）1 and 2 achieved complete remission（CR）and disease stability at 3 months post murine CAR-T therapy. However, 16 and 18 months later, they experienced progression of disease （PD）, and patient 1 presented with extramedullary disease at PD. Both of the patients received humanized CAR-T therapy and achieved partial remission （PR） and very good partial remission （VGPR） post humanized CAR-T therapy. PT1 achieved CR of the soft tissue masses at 4 months post humanized CAR-T therapy. Notably, the median peak of the BCMA CAR-T cells, copy of BCMA CAR gene, persistence of BCMA CAR-T, and the peak levels of IL-6, IL-8, IL-10, IFN-γ and TNF-α were higher in humanized CAR-T therapy than those in the murine CAR-T therapy. During the murine CAR-T therapy, both of the patients experienced grade 1 CRS and no ICANS. PT1 experienced grade 3 CRS and grade 2 ICANS during humanized CAR-T therapy, which were relieved by supportive care. Grade 2 CRS was observed for patient 2 during humanized CAR-T therapy. Humanized BCMA CAR-T cells showed a higher inflammatory response and in vitro cytotoxicity than that of murine BCMA CAR-T cells with effector/targets cells at 1∶1 over 48 hours（*P*<0.001）. The proportions of residual cells in humanized BCMA CAR-T and murine CAR-T were（17.38±5.18）％ *vs*（28.27±4.58）％,（13.25±1.62）％ *vs*（22.77±1.77）％ for PT1 and PT2, respectively.

**Conclusion:**

The humanized BCMA CAR-T cell therapy was efficient and safe for patients who experienced progression of disease after the murine CAR-T therapy, especially for patients with extramedullary disease.

多发性骨髓瘤（MM）是一种浆细胞克隆性增生的恶性肿瘤，呈高度异质性[1]。蛋白酶体抑制剂、免疫调节剂及新药的相继问世已显著改善MM患者的总体生存（OS）期，然而多数患者存在复发进展、难治及耐药的问题，目前MM仍不可治愈。B细胞成熟抗原（BCMA）是治疗MM的有效靶点，BCMA靶向嵌合抗原受体T细胞（CAR-T细胞）治疗复发/难治性MM（RRMM）患者可获得较好的深度缓解，但多数患者最终仍会复发，无进展生存（PFS）期较短[2]–[3]。目前国内外开展的BCMA CAR-T临床试验根据单链抗体可变区片段（scFV）主要分为鼠源及人源化两种。本研究我们对2例鼠源BCMA CAR-T细胞治疗后复发进展的患者予人源化CAR-T细胞治疗，取得较好的疗效，不良反应可控。现总结该两例患者临床特征、CAR-T细胞输注情况及不良反应如下。

## 病例与方法

1. 病例资料：2例RRMM患者均经血常规、血生化、电解质、骨髓病理、血清蛋白电泳、免疫固定电泳、染色体等检查确诊，采用国际骨髓瘤工作组（IMWG）的诊断标准及国际骨髓瘤分期体系（ISS）的分期。例1，女，71岁，2017年10月诊断MM IgG κ型（DS分期Ⅲ期A组，ISS分期Ⅱ期，R-ISS分期Ⅱ期），先后予硼替佐米、环磷酰胺、脂质体阿霉素、来那度胺等药物治疗，以难治MM入组鼠源BCMA CAR-T临床试验（ChiCTR1800017051），鼠源CAR-T治疗前HGB 118 g/L，骨髓涂片骨髓瘤细胞占6％，血清学M蛋白为28.7 g/L。接受FC方案（氟达拉滨30 mg·m^−2^·d^−1^+环磷酰胺400 mg·m^−2^·d^−1^）预处理后，输注4×10^6^/kg自体鼠源BCMA CAR-T细胞。例2，男，61岁，听力障碍者，生活不能自理，2017年8月诊断MM轻链型λ型（DS分期Ⅲ期A组，ISS分期Ⅱ期，R-ISS分期Ⅱ期），M蛋白不可测量。该患者先后接受硼替佐米、沙利度胺、环磷酰胺、来那度胺等药物，以难治MM入组鼠源BCMA CAR-T临床试验（ChiCTR1800017051）。鼠源CAR-T治疗前HGB 109 g/L，骨髓涂片瘤细胞占13.5％，受累与非受累游离轻链比值为差值（dFLC）331 mg/L，λ/κ比值（rFLC）为40，预处理同例1，后输注4×10^6^/kg自体鼠源BCMA CAR-T细胞。本临床试验经我院伦理委员会批准，所有患者签署临床试验知情同意书。

2. CAR-T细胞的制备及质控：本研究鼠源及人源BCMA CAR-T细胞为第二代（共刺激分子为4-1BB）的CAR-T细胞。单采获得患者外周血单个核细胞，CD3磁珠分选T细胞后采用CD3/CD28磁珠（美国Thermo Fisher公司产品）体外扩增T细胞，第4天以鼠源/人源化BCMA CAR质粒（美国Creative Biolabs公司产品）感染扩增的T细胞。12 d后收获，流式细胞术测定鼠源/人源化BCMA CAR的转染效率。质量控制包括生化检测、微生物检测、细胞学检测，均在我中心实验室完成。

3. 疗效评定标准及输注不良反应：疗效标准采用IMWG的疗效标准，即分为完全缓解（CR）、非常好的部分缓解（VGPR）、部分缓解（PR）、疾病稳定（SD）、疾病进展（PD）。总体反应率（ORR）定义为PR及以上。细胞因子释放综合征（CRS）及免疫效应细胞治疗相关神经毒性（ICANS）均参考美国移植及细胞治疗协会标准[4]。CAR-T输注期间不良反应参考CTCAE 5.0版。

4. BCMA-CAR基因扩增及持续时间的监测：CAR-T输注后第0、4、7、14、21、28天，输注后第2～6月每月1次，输注6个月后每3个月1次采用流式细胞术检测外周血BCMA CAR-T扩增；实时定量PCR（q-PCR）检测外周血BCMA CAR DNA拷贝数；酶联免疫吸附试验检测外周血细胞因子IL-6、IL-8、IL-10、IFN-γ及TNF-α变化。

5. 体外杀伤活性及细胞因子检测：培养第12天收获细胞，以MM原代单个核细胞为靶细胞，鼠源/人源化BCMACAR-T细胞、空载体组CD3^+^ T细胞分别作为效应细胞，每组设3个复孔，实验重复3次。效靶比设为1∶1共培养48 h，流式细胞术检测共培养体系中BCMA^+^残余细胞的比例。采用酶联免疫吸附试验检测共培养上清液中细胞因子IFN-γ、TNF-α及IL-6的水平。

## 结果

一、患者临床结果

1. 临床疗效及转归：例1鼠源BCMA CAR-T细胞治疗后3个月血尿免疫固定电泳阴性，骨髓活检示骨髓瘤细胞比例降至0，疗效评估为CR。16个月后临床复发，M蛋白定量为11.2 g/L，予以Rd（来那度胺+地塞米松）方案治疗8个月复查M蛋白定量22.7 g/L，骨髓瘤细胞占10％，HGB 72 g/L，胸部CT示胸椎5～10水平右侧椎旁软组织肿块（图1A），考虑病情进展，且出现髓外病变（EMD）。患者入组人源化BCMA CAR-T临床试验（ChiCTR2000033925）。接受FC方案预处理后输注CAR-T细胞2×10^6^/kg，输注后4个月胸CT示髓外病变示胸5-10水平右侧椎旁软组织肿块消失（图1B），骨髓活检示骨髓瘤细胞降至0，M蛋白定量为6.7 g/L，疗效评估为PR。

**图1:**
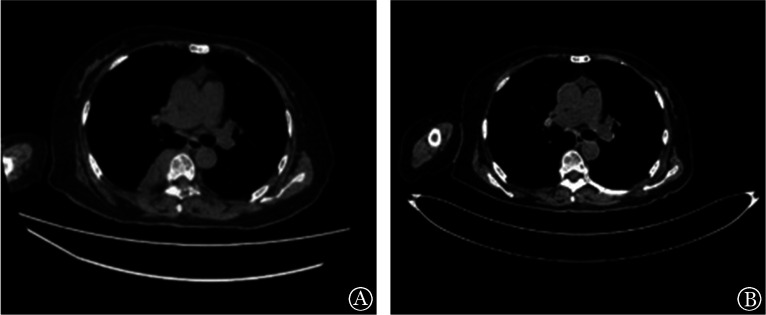
例1人源BCMA CAR-T细胞治疗前（A）及治疗后（B）椎旁软组织肿块变化 BCMA CAR-T细胞：B细胞成熟抗原靶向的嵌合抗原受体T细胞

例2鼠源CAR-T输注3个月后dFLC升至760 mg/L，疗效评估为SD。鼠源CAR-T输注后，患者接受姑息治疗18个月，HGB 68 g/L，dFLC升至3925 mg/L，疾病进展。该患者入组人源化CAR-T临床试验（ChiCTR2000033925），接受FC方案预处理后输注自体CAR-T细胞2×10^6^/kg，输注后3个月后dFLC降至15 mg/L，疗效评估为VGPR。

截止2020年10月，两例患者均存活，例1实际OS期为36个月，例2实际OS期为38个月。

2. CAR-T扩增及持续时间：两次BCMA CAR-T输注剂量及临床疗效见表1。例1输注鼠源CAR-T后第7天时外周血CAR-T达峰值8.6％，CAR-DNA为3.4×10^4^拷贝数/µg，人源化CAR-T输注第14天时外周血CAR-T达峰值48.4％，CAR-DNA为1.9×10^5^拷贝数/µg。例2输注鼠源CAR-T第7天时外周血CAR-T峰值为23.5％，CAR-DNA为9.4×10^4^拷贝数/µg，人源化CAR-T输注第14天扩增达峰值58.3％，CAR-DNA为2.3×10^5^拷贝数/µg。例1输注人源化CAR-T第28天CAR-DNA为2.2×10^4^拷贝数/µg，90 d后CAR-DNA恢复至基线水平；而鼠源CAR-T输注后第28天CAR-DNA恢复到基线水平。例2接受人源化CAR-T输注后第28天时，外周血仍可检测到较高水平的CAR-DNA（1.2×10^5^拷贝数/µg），输注后120 d CAR-DNA恢复至基线水平；而鼠源CAR-T输注期间第28天恢复至基线水平。IL-6、IL-8、IFN-γ、IL-10及TNF-α在鼠源CAR-T及人源化CAR-T输注后第7～14天可检测到峰值，且IL-6、IL-8、IFN-γ、IL-10及TNF-α峰值水平在输注人源化CAR-T期间较鼠源CAR-T水平升高。

**表1:** 两例多发性骨髓瘤患者鼠源及人源化BCMA CAR-T输注情况及疗效

例号	CAR-T回输剂量	CAR-T扩增峰值	CAR-T持续时间	CRS分级	ICANS分级	疗效
1	鼠源：4×10^6^/kg； 人源化：2×10^6^/kg	鼠源：3.4×10^4^拷贝数/µg； 人源化：1.9×10^5^拷贝数/µg	鼠源：28d； 人源化：90d	鼠源：1级； 人源化：3级	鼠源：0级； 人源化：2级	鼠源：CR； 人源化：PR
2	鼠源：4×10^6^/kg； 人源化：2×10^6^/kg	鼠源：9.4×10^4^拷贝数/µg； 人源化：2.3×10^5^拷贝数/µg	鼠源：28d； 人源化：120d	鼠源：1级； 人源化：2级	鼠源：0级； 人源化：0级	鼠源：SD； 人源化：VGPR

注：BCMA CAR-T细胞：B细胞成熟抗原靶向的嵌合抗原受体T细胞；CRS：细胞因子释放综合征；ICANS：免疫效应细胞治疗相关神经毒性；CR：完全缓解；PR：部分缓解；SD：疾病稳定；VGPR：非常好的部分缓解

3. CAR-T治疗不良反应：例1输注人源化CAR-T第5天出现发热（最高达38.7 °C），低血压［78/46 mmHg（1 mmHg＝0.133 kPa）］，予多巴胺升压治疗血压可纠正。患者第5天起患者出现神志恍惚、语言混乱、定向困难，头部CT及MRI未见明显异常，腰穿未见明显异常。患者精神症状持续9 d，予静脉地塞米松减轻神经系统反应后精神症状自行消失。人源化CAR-T输注期间例1的CRS反应为3级，ICANS反应为2级。例2人源化CAR-T输注期间CRS反应为2级，无ICANS发生。两例患者鼠源及人源化CAR-T输注期间，3级以上不良反应为淋巴细胞减少、中性粒细胞减少、血小板减少及贫血。

二、鼠源及人源BCMA CAR-T对原代骨髓瘤细胞体外细胞毒作用

1. 鼠源及人源化BCMA CAR-T的转染效率：例1鼠源和人源化BCMA CAR-T细胞转染率分别为41.38％和45.27％，例2鼠源和人源化BCMA CAR-T转染率分别为43.40％和47.09％。

2. 人源化BCMA CAR-T对MM原代细胞细胞具有显著的细胞毒作用：两例MM患者原代细胞BCMA表达分别为91.89％及81.82％。人源化、鼠源BCMA CAR-T分别与例1共培养，效靶比为1∶1，24 h时肿瘤细胞残余比例分别为（56.42±3.09）％、（58.52±2.24）％，与例2共培养肿瘤细胞残余比例分别为（63.38±3.25）％、（60.92±2.27）％，差异无统计学意义（*P*＝0.242，*P*＝0.126），但两组均高于CD3^+^ T对照组（*P*<0.001）；48 h时人源化与鼠源BCMA CAR-T与例1共培养体系中，肿瘤细胞残余比例分别为（17.38±5.18）％、（28.27±4.58）％，与例2共培养肿瘤细胞残余比例分别为（13.25±1.62）％、（22.77±1.77）％，48 h时人源化BCMA CAR-T细胞对MM原代细胞的细胞毒作用优于鼠源CAR-T细胞，差异有统计学意义（*P*值均<0.001），两组亦高于CD3^+^ T对照组（*P*值均<0.001）。

3. 细胞因子释放：BCMA CAR-T细胞分别与例1、例2原代细胞在效靶比为1∶1时共培养48 h，检测培养体系上清中IFN-γ、TNF-α及IL-6水平，人源化/鼠源BCMA CAR-T同例1共培养48 h IFN-γ、TNF-α及IL-6水平分别为（6566.0±133.3）ng/L对（3221.0±116.9）ng/L、（345.3±14.4）ng/L对（178.8±8.0）ng/L、（552.8±20.4）ng/L对（304.7±13.0）ng/L；同例2共培养48 h IFN-γ、TNF-α及IL-6水平分别为（5052.0±89.3）ng/L对（2629.0±122.7）ng/L、（306.3±8.2）ng/L对（198.5±14.1）ng/L、（262.5±9.5）ng/L对（137.3±11.5）ng/L，差异有统计学意义（*P*值均<0.001）。

## 讨论

2017年FDA及欧盟批准CAR-T细胞用于治疗复发难治急性B淋巴细胞白血病（B-ALL）和B细胞淋巴瘤（B-NHL），使细胞免疫治疗成为当今血液肿瘤治疗的前沿。BCMA在成熟B细胞、正常及恶性浆细胞、浆细胞淋巴样树突状细胞表达，而在初始B细胞、记忆B细胞及正常造血干细胞表面不表达[5]。BCMA CAR-T在RRMM中的疗效较CD19靶点、抗免疫球蛋白轻链靶点疗效显著，使得BCMA成为治疗MM最有效的靶点[6]–[7]。Brudno等[6]在全球首先发起的临床试验纳入24例RRMM患者，其中16例接受高剂量（9×10^6^/kg）CAR-T细胞输注，ORR达81％，CR率达13％。随后国内外开展各项BCMA CAR-T细胞临床试验用于治疗RRMM，ORR达63％～100％，CR率最高可达76％[8]。然而多数患者BCMA CAR-T细胞治疗后出现复发进展，涉及以下三方面因素：①MM细胞表面BCMA抗原表达减弱或丢失；②T细胞自身功能耗竭影响CAR-T细胞扩增；③MM肿瘤微环境改变影响CAR-T疗效[3]。

针对BCMA CAR-T细胞治疗后复发问题，已经有多方面的研究，特别是对RRMM患者BCMA抗原丢失问题的探索。MM多个亚克隆的存在使得接受BCMA CAR-T细胞治疗后出现新的或既往存在的BCMA^−^及BCMA^low^的非优势克隆在免疫细胞治疗后成为优势克隆，从而部分患者可以出现BCMA^−^复发。Cohen等[9]利用人源化BCMA CAR-T治疗RRMM的临床研究中，12/18（67％）患者接受BCMA CAR-T输注后1个月出现残余MM恶性克隆BCMA表达下降，相比于治疗无效的患者，这种现象在有治疗反应的患者中更为明显。另外一项采用人源化BCMA CAR-T的FCARH143临床试验，1例患者BCMA CAR-T治疗后复发时出现BCMA^—^浆细胞群，且残余的BCMA^+^恶性克隆BCMA表达下降70％[3]。本研究中，两例患者接受鼠源CAR-T治疗后MM恶性克隆BCMA表达明显减低，复发进展时部分恶性肿瘤细胞为BCMA^—^细胞群，提示鼠源CAR-T输注后的抗原丢失或表达减弱是引起患者复发进展的主要原因。目前临床有效克服BCMA阴性复发的策略包括：BCMA CAR-T及CD19 CAR-T细胞的续惯输注，开发BCMA在内的双靶点CAR-T细胞等[10]。

诸多临床试验数据表明，CAR-T细胞在体内扩增情况是影响CAR-T治疗后PFS的重要因素之一[6],[11]。首先，T细胞自身功能的耗竭也是影响CAR-T在体内持续扩增的重要因素。研究发现MM患者单采物中CD8^+^初始T细胞或记忆干细胞比例越高，CAR-T输注后疗效越好[9]。在一项纳入17例RRMM患者的LCAR-38临床试验中，研究者发现CAR-T抗体（ADA）阳性患者CAR-DNA扩增峰值与ADA阴性者无差异，但是ADA阳性患者CAR-T在体内持续存在时间较阴性者显著降低，复发前或复发时ADA阳性是影响CAR-T复发的主要危险因素[8]。本研究中例2输注鼠源CAR-T无效，而人源化CAR-T输注有效，考虑与人源化CAR-T在体内持续存在时间较长有关。此外，在人源化CD19 CAR-T临床试验中，研究者发现源自小鼠抗体的抗原识别域具有潜在的免疫原性并可能易被人体免疫排斥，而全人源化的抗原识别域可以降低CAR的免疫原性，人源化CAR-T结合能力较鼠源明显增高，且CAR-T在体内持续时间延长[12]。Turtle等[13]针对复发难治B-ALL研究中，5例首次鼠源CD19 CAR-T治疗后未缓解患者二次予鼠源CD19 CAR-T治疗，CAR-T细胞在体内均未出现扩增峰值及持续扩增，二次鼠源CAR-T细胞治疗无抗肿瘤活性。另外一项在人源化CD19 CAR-T临床试验中，Zhao等[14]发现源自小鼠抗体的抗原识别域具有潜在的免疫原性并可能易被人体免疫排斥，而全人源化的抗原识别域可以降低CAR的免疫原性，人源化CAR-T结合能力较鼠源明显增高，且CAR-T在体内持续时间延长。揭示鼠源治疗无效后二次鼠源治疗无效，而二次人源化CAR-T治疗有效的潜在机制。本研究中，两例患者人源化BCMA CAR-T的扩增峰值及持续时间较鼠源CAR-T有优势，体外试验提示人源化CAR-T较鼠源CAR-T杀伤活性显著增强，初步揭示了二次人源化输注有效的可能机制。

伴有EMD的MM患者通常伴有高的LDH水平，高危细胞遗传学改变或高危基因表达谱的改变，此类患者被视为高危MM[15]。对于伴EMD患者的传统免疫化疗疗效欠佳，OS期较短[15]。硼替佐米可以改善t（4;14）或del（17p）高危MM患者的不良预后，但是对于t（4;14）伴有del（17p）患者无效[16]。泊马度胺对EMD的ORR为30％,达雷妥尤单抗可使部分EMD患者达到PR[17]–[18]。总体而言，新药不能克服伴有EMD患者的不良预后[15]。Cohen等[9]的研究纳入25例RRMM患者，7例是伴有EMD的患者，4例有治疗反应，CAR-T输注后1个月和（或）3个月到达骨髓微小残留病（MRD）阴性，而骨髓达到初始治疗反应的时间为14 d。对于伴有EMD的患者，CAR-T细胞对浆细胞瘤有治疗作用，但其髓外病变清除时间较骨髓MRD转阴时间要长[8]。该研究中伴有EMD的MM患者髓外浸润主要累及皮肤、肝脏、下颚，骨髓MRD在CAR-T输注后短期转阴，而髓外病变肿块是逐步消失的[8]。本研究中，例1疾病进展中出现EMD，人源化CAR-T治疗后4个月椎旁软组织消失。综上，BCMA CAR-T治疗在伴有EMD患者中体现出较好的缓解率，但较骨髓缓解所需时间长，BCMA CAR-T为伴有EMD的高危MM患者提供了新的治疗选择。

CRS及ICANS是BCMA CAR-T治疗过程中的主要不良反应。综合众多BCMA CAR-T临床试验结果，CRS的发生率为60％～100％，ICANS发生率达30％～40％[3]。IL-6、C反应蛋白及铁蛋白峰值水平与CRS、ICNAS发生严重程度正相关[19]。本研究中例1输注人源化CAR-T期间出现3级CRS及2级ICANS反应，较鼠源CAR-T输注期间1级CRS严重，人源化CAR-T输注期间细胞因子IL-6、IL-10及TNF-α峰值水平在也较鼠源CAR-T水平升高，CRS不良反应经过支持对症治疗得到控制，ICANS随CAR-T比例下降自行缓解。

综上，本研究中我们对2例鼠源BCMA CAR-T输注后复发进展的RRMM患者输注人源化CAR-T，显示出一定疗效，且安全性可控。由于病例数量较少，尚需进一步更大样本临床观察。
